# Background Factors Affecting the Radiation Exposure of the Lens of the Eye among Nurses in Interventional Radiology: A Quantitative Observational Study

**DOI:** 10.3390/nursrep14010032

**Published:** 2024-02-10

**Authors:** Tomoko Kuriyama, Takashi Moritake, Koichi Nakagami, Koichi Morota, Go Hitomi, Hiroko Kitamura

**Affiliations:** 1 Department of Occupational and Community Health Nursing, School of Health Sciences, University of Occupational and Environmental Health, Japan, Kitakyushu 807-8555, Japan; kuritomo@med.uoeh-u.ac.jp; 2 Department of Radiation Regulatory Science Research, National Institute of Radiological Sciences, National Institute for Quantum Science and Technology, Chiba 263-8555, Japan; 3 Department of Radiology, Hospital of the University of Occupational and Environmental Health, Japan, Kitakyushu 807-8556, Japan; nakagami@clnc.uoeh-u.ac.jp; 4 Department of Radiology, Shinkomonji Hospital, Kitakyushu 800-0057, Japan; morota@shinkomonji-hp.jp; 5 Department of Radiological Technology, Kawasaki Medical School Hospital, Kurashiki 701-0192, Japan; hitomi@med.kawasaki-m.ac.jp; 6 Occupational Health Training Center, University of Occupational and Environmental Health, Japan, Kitakyushu 807-8555, Japan; h-kita@med.uoeh-u.ac.jp

**Keywords:** interventional radiology, nurse, occupational exposure, radiation protection, work environment, location, staffing level

## Abstract

With the International Commission on Radiological Protection’s (ICRP) reduction in the radiation dose threshold for cataracts, evaluating and preventing radiation exposure to the lens of the eye among interventional radiology (IR) staff have become urgent tasks. In this study, we focused on differences in lens-equivalent dose (H_T Lens_) to which IR nurses in three hospitals were exposed and aimed to identify factors underlying these differences. According to analyses of time-, distance-, and shielding-related factors, the magnitude of the H_T Lens_ dose to which IR nurses were exposed could be explained not by time or shielding but by the distance between the X-ray exposure field and the location of the IR nurse. This distance tended to be shorter in hospitals with fewer staff. The most effective means of reducing the exposure of the lenses of IR nurses’ eyes to radiation is to position them at least two meters from the radiation source during angiography procedures. However, some hospitals must provide IR departments with comparatively few staff. In work environments where it is infeasible to reduce exposure by increasing distance, interventions to reduce time by managing working practices and investment in shielding equipment are also important. This study was not registered.

## 1. Introduction

Interventional Radiology (IR), a diagnostic and treatment technique using fluoroscopy, was proposed by Margulis [1], and Wallace reported its application to the diagnosis and treatment of patients with neoplastic diseases [2]. Recent advances in X-ray-fluoroscopy-unit performance and the development of new tools and devices mean that the applicatory range of IR is now expanding to many body parts, such as those addressed in cardiovascular and cerebrovascular fields. However, its nature as an indirect procedure in which a catheter is guided to the lesion site under fluoroscopic examination using X-rays entails an unavoidable risk of radiation exposure. In particularly difficult treatments, radiation exposure may continue for a long time, as a result of which patients are at risk of developing skin damage such as hair loss and ulceration [3,4,5,6,7]. In addition, some of the radiation directed at the patient changes its direction within the patient’s body, causing medical staff to be exposed to scattered X-rays. Although the exposure of medical staff is substantially lower than that of patients, their cumulative dose is increased by repeated radiological work. To date, there have been no reported cases of skin cancer in medical staff exposed to such low doses of scattered X-rays. However, since carcinogenesis is considered a stochastic effect with no threshold dose, it cannot be reliably stated that there is no risk.

The International Atomic Energy Agency (IAEA) classifies workers involved in IR in medical settings as among “those exposed to highly non-uniform radiation fields in which the lens of the eye may be preferentially exposed”, and for whom it is therefore important to prevent radiation exposure to the lens of the eye [8]. When the eye’s lens is exposed to radiation, opacity appears, and this lens opacity progresses to the point of visual impairment, called a cataract [9,10]. There are three predominant forms of cataracts depending on the location of the cataract: a cortical cataract, a nuclear cataract, and a posterior subcapsular cataract (PSC) [11]. A PSC is considered a characteristic finding of radiation cataracts [12,13,14]. Cataracts may also occur due to age [15], UV exposure [16,17,18,19], corticosteroid medication [20,21], and diabetes mellitus [22]. In six countries where differences in healthcare systems may lead to differences in eye exposure levels, the Retrospective Evaluation of Lens Injuries and Dose (RELID) study was conducted under the auspices of the IAEA in 2008, finding that approximately 40–50% of interventionists and 20–40% of technicians or nurses had posterior subcapsular opacities consistent with injuries derived from exposure to ionizing radiation [23,24,25,26,27]. The International Commission on Radiological Protection (ICRP) issued a recommendation in 2011 to reduce the threshold dose for cataracts from 8 Gy to 500 mGy [14]. Since then, many countries have lowered their lens-equivalent dose limits, and in April 2021, Japan also reduced this limit from 150 mSv to 20 mSv per year [28].

Among IR staff, physicians have the highest radiation exposure levels, with the exposure level for nurses being reportedly less than a third of that of physicians [29,30,31,32]. Some studies examining the causes of radiation exposure among IR nurses and its background factors have found that these causes are not the same as those of physicians [33] and that there was no correlation between the doses administered to the primary operator and those applied to IR nurses providing direct or indirect assistance [31], pointing out that the different roles of physicians and nurses working in IR may affect the radiation dose [31]. However, an analysis that depends solely on simple comparisons with physicians at a single site is not sufficient. In this study, we selected three Japanese hospitals of different sizes and compared the levels of exposure of the lens the eye to radiation among IR nurses working in these institutions. We also analyzed factors that might affect the dose received by IR nurses’ eye lenses from the perspective of the three relevant concepts of time, distance, and shielding in order to keep the radiation dose administered to medical professionals as low as reasonably achievable [34,35].

## 2. Materials and Methods

### 2.1. Study Design

This was a quantitative observational study of the lens-equivalent doses (H_T Lens_) to which IR nurses were exposed.

### 2.2. Study Sites and Data Gathered

In Japan, hospitals with 200 or more beds account for over 60% of hospitals with angiography equipment [36]. For this study, we selected three designated emergency hospitals, all with over 200 beds but of different functionalities and sizes (Hospital A, with 678 beds; Hospital B, with 1182; and Hospital C, with 214). Hospitals A and B were both university hospitals, and Hospital C was a private hospital. 

The study period was from January 2018 to March 2021. We analyzed a total of 3371 consecutive cases (Hospital A, *n* = 900; Hospital B, *n* = 1979; Hospital C, *n* = 492) during a one-year period set individually by each hospital (Hospital A, April 2020–March 2021; Hospital B, February 2020–January 2021; Hospital C, January 2018–December 2018).

**Figure 1:**
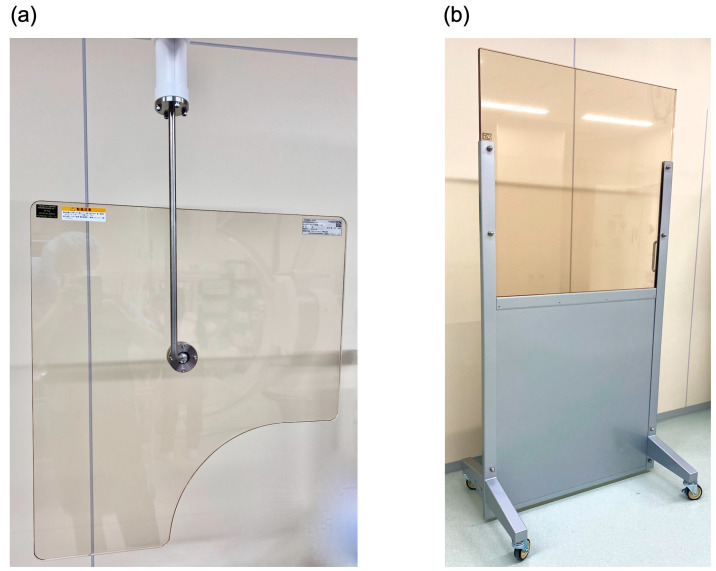
Lead lined radiation-shielding equipment installed in angiography rooms: (**a**) Ceiling-suspended lead shield (CSS) and (**b**) rolling lead shield (RS). The thickness of both is 0.5 mm Pb equivalent.

This study included 88 IR nurses (Hospital A, *n* = 31; Hospital B, *n* = 49; Hospital C, *n* = 8), excluding those for whom individual radiation dose information was not reported for 1 month or more for reasons such as maternity leave, and the total lens-equivalent dose (H_T Lens total_) for each participant was calculated. Regarding the concepts of time, distance, and shielding, for time, we compiled the total number of cases for which each IR nurse had been responsible during the year (C_total_); the number of these cases in which IR had been therapeutically conducted, therefore requiring higher X-ray doses than IR for diagnostic purposes (C_therapeutic_); total fluoroscopy time (FT_total_); and total air kerma-area product (P_KA total_). As distance-related data, we compiled information on the equipment layout in the angiography rooms used and the locations of the IR nurses. For shielding, we gathered information on the use of ceiling-suspended lead shields (CSS; Figure 1a) and rolling lead shields (RS; Figure 1b) fitted in the angiography room, which may affect the H_T Lens total_ of IR nurses. We also recorded the number of physicians and nurses who were employed in the treatment of each IR patient. These numbers included standby staff.

### 2.3. Lens-Equivalent Dose Measurements

IR nurses wore protective lead aprons to which they attached two individual dosimeters, one to the inside of the lead apron and another to the outside at the top of the collar. The individual dosimeters used at Hospitals A and B were radio-photoluminescence dosimeters (Chiyoda Technol Corporation, Tokyo, Japan), and those used at Hospital C were optically stimulated luminescence dosimeters (Nagase-Landauer, Ltd., Tsukuba, Japan). The 10 mm individual dose equivalent [Hp (10)] measured by the individual dosimeter on the outside of the lead apron was calculated as the H_T Lens_. The integrated value for each month was measured at a detection threshold of 0.05 mSv and rounded up or down, and values of ≥0.1 mSv were reported in 0.1 mSv increments. All values below 0.05 mSv were reported as 0 mSv.

### 2.4. Mean Distance between Station and X-ray Irradiation Field

For each angiography room, we investigated the size of the room, its equipment layout, and the distance between the center of rotation of the C-arm on the angiography unit (regarded as the X-ray irradiation field) and the position where the IR nurse was stationed for the longest time, for example, when completing nursing records. The weighted mean distance between the X-ray irradiation field and the IR nurse’s station (D_mean_) was calculated for each IR nurse using Equation (1).

D_mean_ (cm) = Σ(D_i_ × C_i_/C_total_) (1)

Here, D_i_ is the distance (cm) between the X-ray irradiation field and the station in Room_i_, C_i_ is the number of cases for which an IR nurse was responsible in Room_i_ during the year, and C_total_ is the total number of cases for which an IR nurse was responsible.

### 2.5. Lens-Equivalent Dose Rate

The H_T Lens_ of IR nurses is directly affected by the number of times they are engaged in IR and for how long. To eliminate this effect, we evaluated the lens-equivalent dose ratio (H_T_R_Lens_) for each IR nurse using Equation (2).

H_T_R_Lens_ (μSv/h) = H_T Lens total_/FT_total_ (2)

FT_total_ is the total fluoroscopy time for the cases for which an IR nurse was responsible during the year.

### 2.6. Distance between the X-ray Irradiation Field and the Station and Its Relationship with IR Staff Numbers

We calculated the mean total number of physicians and IR nurses (IR staff) other than clinical radiologists who worked on one IR procedure (S_mean_) for each IR nurse using Equation (3).

S_mean_ = S_total_/C_total_ (3)

S_total_ is the total number of IR staff who worked on all the cases for which an IR nurse was responsible during the year. We then analyzed the association between S_mean_ and D_mean_.

### 2.7. IR Staffing Levels

We categorized the number of physicians and IR nurses who worked on one IR procedure as the following four categories: 1, 2, 3, or ≥4 members of staff.

### 2.8. Statistical Methods

We analyzed the relationship between the parameters H_T Lens total_, C_total_, C_therapeutic_, FT_total_, and P_KA total_ for IR nurses and the various factors by calculating Spearman’s rank correlation coefficient (*ρ*). Multiple comparisons of factors between the different hospitals were carried out by conducting a Kruskal–Wallis test followed by a Mann–Whitney *U* test (Bonferroni-adjusted for double testing). To analyze the correlation between S_mean_ and D_mean_, Pearson’s correlation coefficient (*r*) and Spearman’s rank correlation coefficient (*ρ*) were calculated. Correlation coefficient values *r* or *ρ* less than 0.4 represent a weak correlation, values ranging from 0.4 to 0.69 represent a moderate correlation, and values ranging from 0.70 to 0.99 represent a strong correlation [37,38]. Multiple comparisons of the total numbers of physicians and IR nurses between the different hospitals were carried out by conducting a Kruskal–Wallis test followed by a Mann–Whitney *U* test (Bonferroni-adjusted for double testing). SPSS Ver. 25 for Windows (SPSS Inc., Chicago, IL, USA) and OriginLabs OriginPro2023 (OriginLab Corporation, Northampton, MA, USA) were used for analysis, and all *p*-values were two-tailed. Statistical significance was set at *p* < 0.05.

## 3. Results

### 3.1. Comparison of H_T Lens total_ among Hospitals

At Hospital C, none of the IR nurses had an H_T Lens total_ of 0 mSv, but at both Hospitals A and B, 61% of IR nurses had an H_T Lens total_ of 0 mSv. Moreover, IR nurses at Hospital C had a higher mean H_T Lens total_ than those at Hospitals A and B (Table 1). An analysis restricted to only those nurses whose H_T Lens total_ values were ≥ 0.1 mSv showed that the H_T Lens total_ values differed significantly among the hospitals (Kruskal–Wallis test, *p* < 0.01). According to the results of multiple comparison testing, H_T Lens total_ was significantly higher at Hospital C than at Hospitals A and B (Mann–Whitney *U* test, *p* < 0.05; Table 1). No nurses at any of the three hospitals exceeded the lens-equivalent dose limit (20 mSv/year).

### 3.2. Associations between H_T Lens total_ and Time-Related Factors

**Figure 2:**
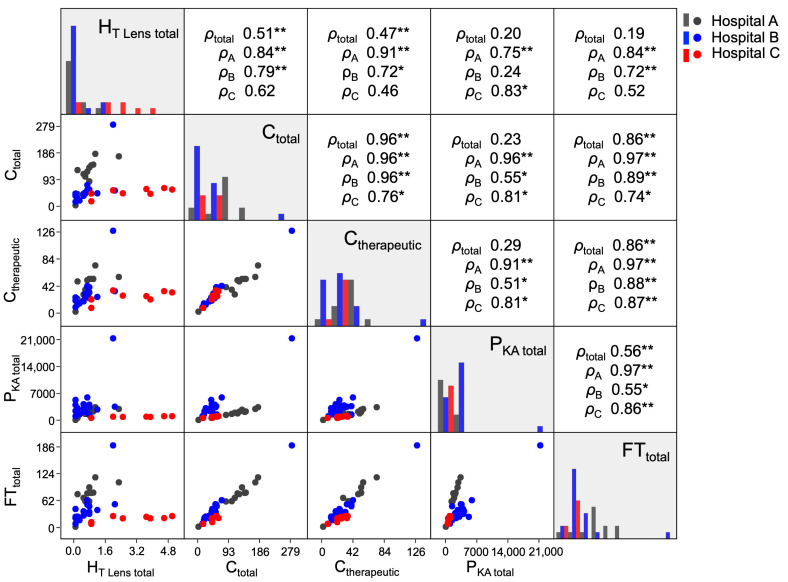
Relationships between the total lens-equivalent dose delivered to IR nurses and their total number of cases, total number of therapeutic IR cases, total air kerma-area product, and fluoroscopy time. The analyzed population comprised 39 nurses whose H_T Lens total_ values were ≥0.1 mSv. The lower-left triangular matrix shows scatter plots, the squares on the diagonal show histograms, and the upper right triangular matrix shows the correlation coefficients. Spearman’s rank correlation coefficients *ρ*_total_, *ρ*_A_, *ρ*_B_, and *ρ*_C_ are those for all three hospitals together and those for individual analyses of Hospitals A, B, and C, respectively. * *p* < 0.05; ** *p* < 0.01.

Figure 2 shows a scatter plot matrix of the associations between H_T Lens total_, C_total_, C_therapeutic_, FT_total_, and P_KA total_. An analysis of how the four time-related parameters of C_total_, C_therapeutic_, FT_total_, and P_KA total_ were associated with each other at each hospital showed a moderate to strong correlation between every combination (0.51 ≤ *ρ*_A/B/C_ ≤ 0.97). Similarly, the results of an analysis of all three hospitals together showed strong correlations among three parameters, namely, C_total_, C_therapeutic_, and FT_total_ (0.86 ≤ *ρ*_total_ ≤ 0.96); however, they showed weak to moderate correlations between P_KA total_ and these three parameters (0.23 ≤ *ρ*_total_ ≤ 0.56). In the analysis of all three hospitals together, H_T Lens total_ was weakly to moderately correlated with all time-related parameters (0.19 ≤ *ρ*_total_ ≤ 0.51), and in an analysis of Hospital A alone, these correlations were particularly strong (0.75 ≤ *ρ*_A_ ≤ 0.91).

### 3.3. Equipment of Angiography Rooms with Radiation Shields

**Figure 3:**
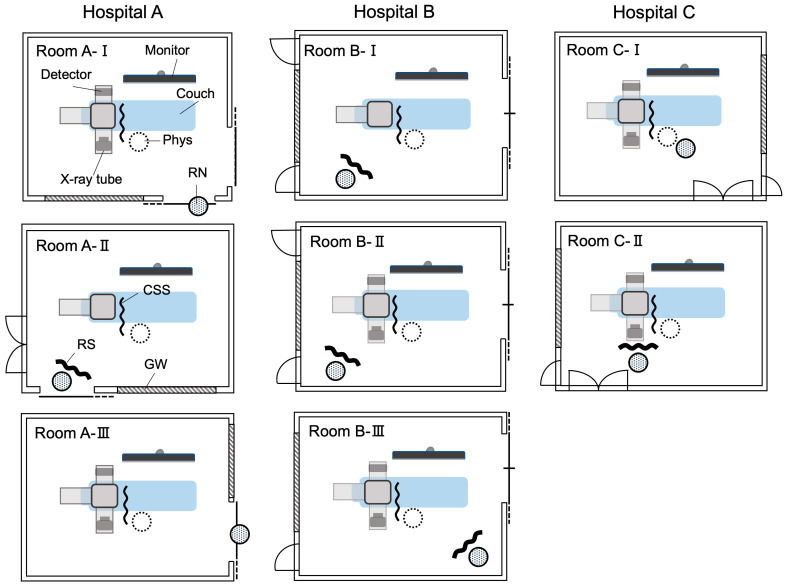
Floor plans of angiography rooms. GW, lead-containing glass window between angiography room and control room; Phys, position of the first physician; RN, position of the IR nurse.

In terms of shielding, RSs were installed in the angiography rooms in Hospital B (Table 2), and the IR nurses conducted their tasks while stationed behind them (Figure 3). At Hospital A, RSs were not installed in Room A-I and Room A-III (Table 2); in these two rooms, the IR nurse stood outside at a distance from the X-ray irradiation field and carried out most of their tasks in a position in which they were behind the door of the angiography room (Figure 3). At Hospital C, an RS was not installed in Room C-I, but most of the patients underwent procedures in Room C-II, which was fitted with an RS (Table 2). CSSs were fitted in all the rooms of all the hospitals and were properly used (Table 2).

### 3.4. Association between H_T Lens total_ and X-ray Irradiation Field–Station Distance

**Figure 4:**
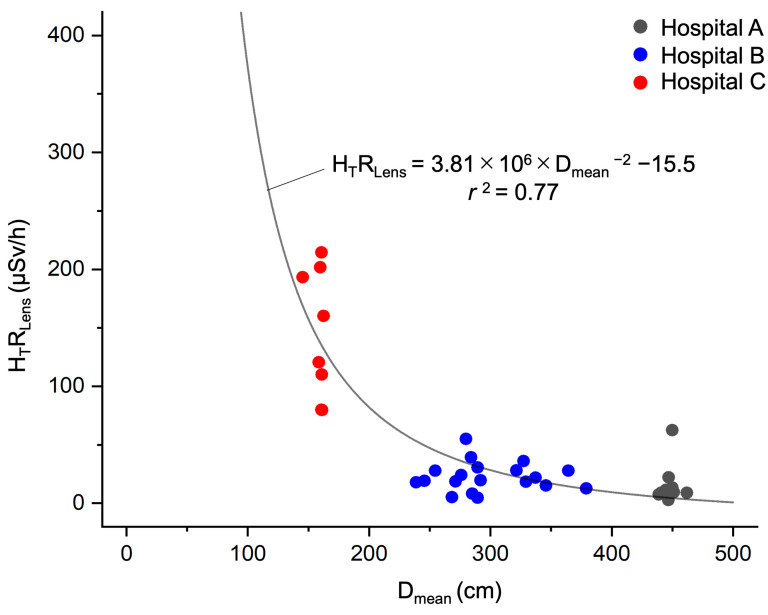
Association between lens-equivalent dose rate and mean distance between X-ray irradiation field and position of the IR nurse. The analysis population comprised 39 nurses whose H_T Lens total_ values were ≥0.1 mSv. The curve was fitted using the least-mean-squares method. D_mean_, mean distance between the X-ray irradiation field and the position of the IR nurse (cm); H_T_R_Lens_, lens-equivalent dose rate (μSv/h); *r*^2^, coefficient of determination.

To identify factors that may have contributed to the differences in the H_T Lens total_ values between hospitals, we plotted the relationship between the H_T_R_Lens_, which eliminates the effect of time from H_T Lens total_, and D_mean_, and we obtained a good fit (Figure 4) using Equation (4).

H_T_R_Lens_ (μSv/h) = 3.81 × 10^6^ × {D_mean_ (cm)}^−2^ − 15.5 (4)

### 3.5. Association between X-ray Irradiation Field–Station Distance and Number of IR Staff

**Figure 5:**
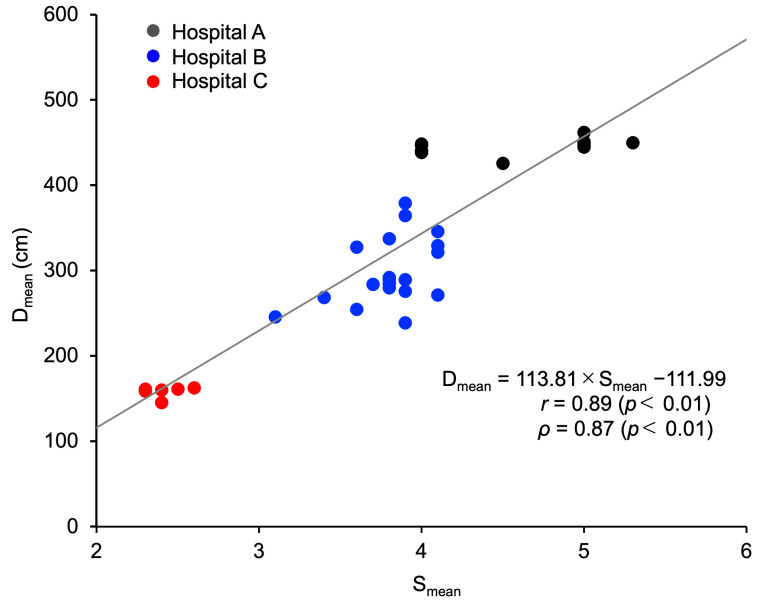
Association between the mean number of IR staff and mean distance between X-ray irradiation field and position of IR nurse. The analysis population comprised 39 nurses whose H_T Lens total_ values were ≥0.1 mSv. *r*, Pearson’s correlation coefficient; S_mean_, mean number of IR staff excluding radiologic technologists; *ρ*, Spearman’s rank correlation coefficient. The line is the regression line.

To explore the reasons underlying the differences in D_mean_, we analyzed its association with S_mean_ and identified a strong positive correlation between the two (*r* = 0.89, *ρ* = 0.87, *p* < 0.01; Figure 5).

### 3.6. Number of IR Staff and IR Staffing Levels

**Figure 6:**
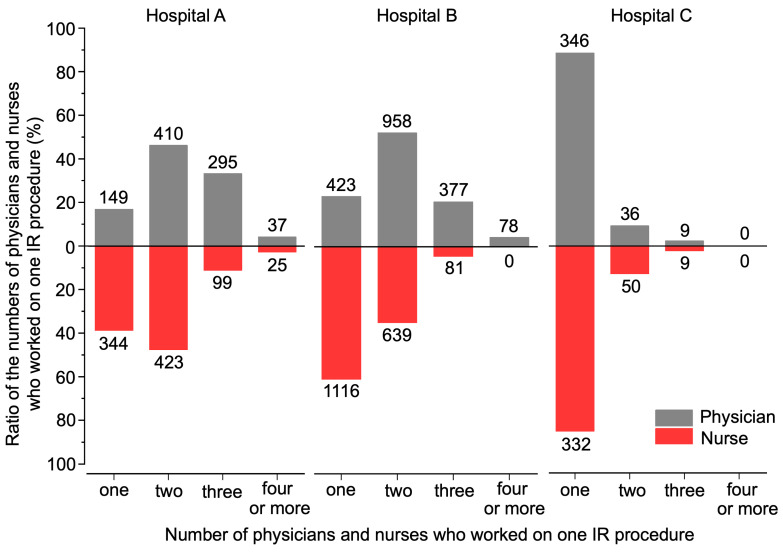
The number of physicians and IR nurses working on IR procedures. The horizontal axis shows the number of physicians and IR nurses working on one IR procedure (1, 2, 3, or ≥4), and the vertical axis indicates the proportion. The numbers at the top of each bar are the numbers of cases.

To explore the reasons for the underlying differences in S_mean_, we investigated the number of IR staff who worked on one IR procedure. Of the total of 3371 cases, 3118 cases were analyzed, wherein the number of both physicians and nurses were available; the number of IR staff differed significantly among the hospitals (Kruskal–Wallis test, *p* < 0.01; Table 3). According to the results of multiple comparison testing, the number of IR staff was significantly smaller at Hospital C than at Hospitals A and B (Mann–Whitney *U* test, *p* < 0.01; Table 3). The most common staff compositions in each hospital were two physicians and two IR nurses at Hospital A, two physicians and one IR nurse at Hospital B, and one physician and one IR nurse at Hospital C (Figure 6).

## 4. Discussion

From the perspective of occupational health and safety, risk areas (in this study, radiation-controlled areas) and areas where people are present must be kept completely separate, either through distancing or by making ingress impossible [39]. For medical staff involved in IR, however, working in radiation-controlled areas is unavoidable, and as the angiography room contains localized spots with high air dose rates, it is necessary to assess the exposure statuses of IR nurses and optimize their protection against radiation even though their exposure doses are usually lower than those of doctors and never exceed the dose limit. In the course of this study, we noticed that the yearly H_T Lens total_ of IR nurses differed among the investigated hospitals, and in light of the possibility that this might be due to hospital-specific factors, we investigated the factors potentially affecting the H_T Lens total_ of IR nurses based on the three concepts of time, distance, and shielding.

An analysis of all 88 IR nurses, including those for whom the H_T Lens total_ was 0 mSv, showed that the highest H_T Lens total_ (mean 2.9 mSv) for IR nurses was at Hospital C. This was also true when nurses with an H_T Lens total_ of 0 mSv were excluded, and the mean value at Hospital C was significantly higher than the same values at Hospitals A and B (Table 1). The correlation analyses showed moderate to strong positive correlations between combinations of the four time-related parameters of C_total_, C_therapeutic_, FT_total_, and P_KA total_ (0.51 ≤ *ρ*_A/B/C_ ≤ 0.97, Figure 2), which can be described as a valid result. At Hospital A, H_T Lens total_ was strongly positively correlated with the four time-related factors (0.75 ≤ *ρ*_A_ ≤ 0.91, Figure 2), and it may be possible to estimate the H_T Lens total_ of IR nurses by combining the values of the time-related parameters. Several studies have reported that FT and P_KA_, which are displayed on the angiography unit as a proxy for patient exposure dose, are generally positively correlated with the radiation dose delivered to the lens of the eye among medical staff [40,41,42,43], but these studies concerned the radiation dose delivered to the lens of the eye among physicians. One study also reported that because IR nurses move around the room, the radiation dose delivered to the lens of the eye among these nurses is not significantly associated with FT or P_KA_ [43]. Our study results also indicate that although the mean values of the four time-related factors at Hospital C were markedly lower than those at Hospitals A and B, H_T Lens total_ was statistically significantly higher at Hospital C than at the other two hospitals (Table 1). This high value of H_T Lens total_ at Hospital C cannot be explained solely in terms of time-related factors, suggesting that hospital-specific factors may be involved.

CSSs are mainly effective as protection for physicians. They were installed in all angiography rooms and properly used, with no evident differences among hospitals (Table 2). RSs, however, are mainly effective as protection for IR nurses. As shown in Figure 3, RSs were not installed in two rooms of Hospital A (Rooms A-I and A-III), where the nurse was positioned outside the door, and in one room of Hospital C (Room C-I), where the IR nurse was positioned immediately behind and to the right of the physician. Although we did not carry out a quantitative investigation of the effect of shielding factors on H_T Lens total_, because the IR nurse was positioned outside the door of Rooms A-I and A-III, the shielding effect would probably have been at least equivalent to that of an RS, and in Room C-I, the physician’s trunk should also have provided a shielding effect. In addition, at Hospital C, approximately two-thirds of the procedures on which IR nurses worked were conducted in Room C-II, which was equipped with an RS (Table 2). Thus, it at least cannot be said with certainty that inadequate shielding at Hospital C was the reason for its higher H_T Lens total_, suggesting that other factors may have been involved. Because we estimated the H_T Lens_ values of IR nurses from the values recorded by individual dosimeters attached to the tops of their collars on the outside of their lead aprons, the use or lack thereof of lead protective glasses did not affect H_T Lens total_.

Distance is a valuable tool for providing radiological protection. Radiation doses decrease with the square of the distance between the radiation source and the operator (according to the inverse-square law). Thus, the dose decreases rapidly when a person moves away from an X-ray source [44]. Because IR nurses move around the angiography room during procedures, it is difficult to accurately measure the distance between them and the patient or X-ray tube [45]. In this study, when we analyzed the association between D_mean_ and H_T_R_Lens_ for each IR nurse on the assumption that their position in each IR room was the location in which they spent the most time, we found that the D_mean_ at Hospital C was much shorter compared to that at Hospitals A and B, and the H_T_R_Lens_ of IR nurses fit well with the inverse square of the distance (Figure 4). This suggests that distance was the main factor underlying the large difference in H_T Lens total_ among the three hospitals in this study. As shown in Figure 4, the slope of the regression curve flattened out when D_mean_ exceeded 2 m, indicating that positioning IR nurses at least 2 m from an X-ray irradiation field is an efficient means of reducing H_T_R_Lens_.

What might be the reason for the difference in D_mean_ among the three hospitals? We first considered the physical size of the angiography rooms, but as shown in Table 2, the floor space did not considerably differ among the three hospitals. In fact, the two angiography rooms at Hospital C were more spacious than those at Hospitals A and B, so the shorter D_mean_ at Hospital C was not because its rooms were smaller. We then looked at the association between D_mean_ and S_mean_, and we found a strong positive correlation between these two parameters (Figure 5), indicating that when fewer IR staff were present, IR nurses tended to work at a closer distance to the X-ray irradiation field. A further investigation of the number of IR staff working on one procedure found that the mean quantities of IR staff were 4.1, 3.5, and 2.3 at Hospitals A, B, and C, respectively (Table 3), and that treatment was usually carried out by two physicians at Hospitals A and B, but at Hospital C, usually only one physician was responsible (Figure 6). Because training residents and medical students is a major part of the work of university hospitals such as Hospitals A and B, the inclusion of residents and others acting as assistants means that the number of physicians involved in treatment tends to be relatively higher. At Hospital C, in which most procedures were conducted by a single physician, there was greater pressure on IR nurses to act as assistants for procedures performed by physicians, and as a result, these IR nurses spent a longer time positioned right next to the physician. IR nurses, including trainee nurses, act as circulating nurses (responsible for tests, preparing the treatment environment, assisting the patient, and recording information) and as scrub nurses (directly assisting the physician and readying equipment) during procedures, and as these tasks directly affect where IR nurses are stationed in the angiography room, they may also influence their exposure doses [46,47,48]. Although this study did not investigate the specific roles played by IR nurses during procedures, it can be conjectured that the roles of IR nurses varied among the participating hospitals.

Unlike physicians and radiologic technicians, who have control over radiation emissions [49], IR nurses may not be aware that their bodies are being exposed and have difficulty taking preventive action against anticipated exposure. Under these circumstances, reducing their exposure cannot depend solely on how aware individual IR nurses are of radiation protection, nor on the wearing of individual protective equipment, which should be the final option [50]. The primary task in this regard is to re-evaluate the stationing of IR nurses and, if possible, instruct them to stand at a distance of at least 2 m from the X-ray irradiation field. However, depending on the circumstances of the hospital, it may be necessary to conduct IR procedures with a small number of staff. In this case, rather than depending entirely on instructing individual IR nurses on how to act, interventions will be required from both the time perspective, e.g., managing working practices so as not to depend too much on a limited number of IR nurses, and from the shielding perspective in terms of facilities investment for installing appropriate shielding equipment such as RSs and CSSs.

This study has several limitations. Although we analyzed time, distance, and shielding as important factors affecting the exposure doses delivered to IR nurses, other factors are also known to influence these doses, including the difficulty of a procedure, the X-ray collimation range, changes in X-ray beam direction, and differences in patient physique [51,52]. Further studies of these factors are required.

## 5. Conclusions

In this study, we found that the exposure dose delivered to the lens of the eye among IR nurses in one hospital was high, although the values of the four time-related factors C_total_, C_therapeutic_, F_total_, and P_KA total_, on which the dose delivered to the lens of the eye depends, were rather low. This was because of a distance-related factor, namely, the distance between the X-ray irradiation field and the location of the nurse. When the circumstances of a hospital dictate that IR procedures must be conducted by a small group of staff, particularly when this group comprises just one physician and one IR nurse, the distance between the X-ray irradiation field and the nurse’s location tends to be substantially shorter. In this case, the nurse should be instructed to stand at least 2 m away from the X-ray irradiation field if possible, and every effort should be made to arrange shift patterns so as not to depend too much on a limited number of IR nurses and to ensure the appropriate use of protective equipment such as RSs and CSSs. This study also showed that the lens equivalent dose for IR nurses was well below the dose limit, so wearing lead protective glasses is not mandatory. However, monitoring the lens dose delivered outside the lead apron is necessary to ensure the dose is zero or very low.

## Figures and Tables

**Table 1:** Interhospital comparisons of total lens-equivalent doses delivered to IR nurses and dose parameters.

Hospital		A			B			C			Interhospital Comparisons ^‡^	
H_T Lens total_ Dose Category		≥0.1 mSv and 0 mSv ^†^	≥0.1 mSv	0 mSv ^†^	≥0.1 mSv and 0 mSv ^†^	≥0.1 mSv	0 mSv ^†^	≥0.1 mSv and 0 mSv ^†^	≥0.1 mSv	0 mSv ^†^	*p* (Kruskal–Wallis)	Multiple Comparison (Mann–Whitney *U*)
Number (%) of IR Nurses		31 (100)	12 (39)	19 (61)	49 (100)	19 (39)	30 (61)	8 (100)	8 (100)	0 (0)		
H_T Lens total_ (mSv)												
	mean ± SD	0.3 ± 0.5	0.8 ± 0.6	N.A.	0.3 ± 0.5	0.7 ± 0.6	N.A.	2.9 ± 1.5	2.9 ± 1.5	N.A.	<0.01	A < C * B < C **
	median [IQR]	0.0 [0.0–0.6]	0.8 [0.4–0.9]	N.A.	0.0 [0.0–0.3]	0.6 [0.2–0.8]	N.A.	3.1 [1.7–4.1]	3.1 [1.7–4.1]	N.A.		
C_total_												
	mean ± SD	50.8 ± 59.2	114.1 ± 49.1	10.8 ± 7.7	49.4 ± 46.3	55.2 ± 55.8	45.7 ± 38.7	48.0 ± 13.7	48.0 ± 13.7	N.A.	<0.01	B < A **
	median [IQR]	16.0 [6.5–107.5]	123.0 [98.8–142.8]	8.0 [4.5–15.0]	44.0 [23.0–56.0]	44.0 [36.0–54.5]	37.0 [19.5–58.5]	50.0 [43.8–58.3]	50.0 [43.8–58.3]	N.A.		
C_therapeutic_												
	mean ± SD	18.4 ± 22.8	43.2 ± 18.0	2.8 ± 3.4	28.5 ± 23.8	30.1 ± 24.9	27.5 ± 23.0	25.5 ± 8.3	25.5 ± 8.3	N.A.	<0.05	B < A *
	median [IQR]	3.0 [1.0–38.0]	50.0 [34.3–53.0]	1.0 [1.0–3.0]	23.0 [15.0–36.0]	24.0 [18.5–33.0]	22.0 [11.0–39.0]	26.5 [21.0–32.5]	26.5 [21.0–32.5]	N.A.		
P_KA total_ (10^3^ × Gycm^2^)												
	mean ± SD	840.0 ± 1013.7	1895.5 ± 901.5	173.1 ± 124.1	3327.3 ± 3418.9	4118.6 ± 4221.4	2826.1 ± 2675.6	819.9 ± 177.8	819.9 ± 177.8	N.A.	<0.01	A < B * C < B **
	median [IQR]	260.5 [102.5–1665.1]	2028.1 [1539.0–2390.2]	115.7 [82.8–248.2]	2823.6 [1451.2–3786.0]	3364.7 [2298.7–3967.8]	2435.3 [1157.2–3572.7]	859.9 [732.1–939.2]	859.9 [732.1–939.2]	N.A.		
FT_total_ (hour)												
	mean ± SD	30.7 ± 36.4	69.5 ± 30.6	6.1 ± 4.4	30.7 ± 31.3	42.2 ± 37.0	23.4 ± 24.5	20.1 ± 6.1	20.1 ± 6.1	N.A.	<0.01	C < A**
	median [IQR]	8.4 [3.9–63.9]	77.2 [60.1–83.0]	4.4 [3.3–8.4]	24.5 [16.7–36.8]	32.7 [24.5–45.2]	18.4 [8.0–30.2]	21.1 [18.6–24.8]	21.1 [18.6–24.8]	N.A.		

H_T Lens_: Lens-equivalent dose. The dose calculated for the lens of the eye, based on the physical dose delivered to the lens, was adjusted to account for the effectiveness of the type of radiation. H_T Lens total_: total lens-equivalent dose. C_total_: total number of cases. C_therapeutic_: number of therapeutic IR cases. P_KA_: Air kerma-area product, i.e., the integral of air kerma across the entire X-ray beam emitted from an X-ray tube. P_KA total_: total air kerma-area product. FT: fluoroscopy time, i.e., moment at which the X-ray beam is emitted from the fluoroscopy system. FT_total_: total fluoroscopy time. SD: Standard deviation. IQR: Interquartile range [1st–3rd quartile]. N.A.: not available, * *p* < 0.05, ** *p* < 0.01, and ^†^ below detection threshold. ^‡^ Analysis was limited to nurses whose H_T Lens total_ values were ≥0.1 mSv.

**Table 2:** Overview of angiography rooms, number of cases conducted during the study period, floor space, distance, and shield installation.

Room	Angiography System	Main Area	Number of Cases	Floor Space (cm^2^)	Distance * (cm)	CSS	RS
Hospital A							
A-I	Alphenix INFX-8000V ^a^	Cerebral	357	4140	500	+	−
A-II	C vision PLUS ^b^	Abdominal	120	4225	400	+	+
A-III	Artis-Zee ^c^	Coronary	423	4225	400 ^†^/350 ^‡^	+	−
Hospital B							
B-I	Alphenix INFX-8000V ^a^	Thoracoabdominal	561	4200	220	+	+
B-II	Allura Xper FD20/10 ^d^	Cerebral	521	4200	220	+	+
B-III	Allura Clarity FD10/10 ^d^	Coronary	897	4200	400	+	+
Hospital C							
C-I	BRANSIST Safire VC9 slender ^b^	Cerebral	169	5016	180	+	−
C-II	BRANSIST Safire ^b^	Coronary	323	5016	150	+	+

* Distance from the center of rotation of the C-arm (X-ray irradiation field) to the location where the IR nurse recorded information (position of the IR nurse). ^†^ Brachial artery approach. ^‡^ Femoral artery approach ^a^ Canon Medical System Corporation, Askim, Sweden. ^b^ Shimadzu Corporation, Kyoto, Japan. ^c^ Siemens Healthineers Solutions, Erlangen, Germany. ^d^ Philips Healthcare, Best, The Netherlands. All devices ^a–d^ were under-tube types, with the X-ray tube situated under the table.

**Table 3:** Inter-hospital comparison of total number of physicians and IR nurses who worked on one IR procedure.

Hospital	A	B	C	Interhospital Comparisons	
Number of Cases ^†^	891	1836	391	*p* (Kruskal–Wallis)	Multiple Comparison (Mann–Whitney *U*)
mean ± SD	4.1 ± 1.3	3.5 ± 1.1	2.3 ± 0.7	<0.01	B < A ** C < A ** C < B **
median [IQR]	4 [3–5]	3 [3,4]	2 [2,2]		

^†^ Of the total 3371 cases, 3118 cases were analyzed, excluding the cases with deficiencies in either the number of physicians or IR nurses or both. SD: Standard deviation. IQR: Interquartile range [1st–3rd quartile]. ** *p* < 0.01

## Data Availability

No new data were created or analyzed in this study. Data are contained within the article.

## Public Involvement Statement

There was no public involvement in any aspect of this research.

## Guidelines and Standards Statement

This manuscript was drafted against the STROBE guidelines for a cross-sectional study.
